# Association of dengue infection with anti-alpha-gal antibodies, IgM, IgG, IgG1, and IgG2

**DOI:** 10.3389/fimmu.2022.1021016

**Published:** 2022-10-14

**Authors:** Olayinka M. Olajiga, L. Paulina Maldonado-Ruiz, Soheila Fatehi, Jenny C. Cardenas, Maria U. Gonzalez, Lady Y. Gutierrez-Silva, Berlin Londono-Renteria, Yoonseong Park

**Affiliations:** ^1^ Department of Entomology, Kansas State University, Manhattan, KS, United States; ^2^ Laboratorio Clínico, Hospital Local Los Patios, Los Patios, Norte de Santander, Colombia; ^3^ Laboratorio Clinico, Empresa Social Del Estado Hospital Emiro Quintero Cañizares, Ocaña, Norte de Santander, Colombia; ^4^ Laboratorio Clínico, Hospital Erasmo Meoz, Cúcuta, Norte de Santander, Colombia; ^5^ School of Public Health and Tropical Medicine, Tulane University, New Orleans, LA, United States

**Keywords:** Alpha-gal (α -gal), dengue (DENV), antibodies, flaviviral infection, vector-borne disease (VBD)

## Abstract

Dengue virus (DENV) transmitted by the *Aedes* mosquitoes is the etiological agent of dengue fever, one of the fastest-growing reemerging mosquito-borne diseases on the planet with a 30-fold surge in the last five decades. Interestingly, many arthropod-borne pathogens, including DENV type 2, have been reported to contain an immunogenic glycan galactose-alpha1,3-galactose (alpha-Gal or aGal). The aGal molecule is a common oligosaccharide found in many microorganisms and in most mammals, except for humans and the Old-World primates. The loss of aGal in humans is considered to be an evolutionary innovation for enabling the production of specific antibodies against aGal that could be presented on the glycan of pathogens. The objective of this study was to evaluate different anti-aGal antibodies (IgM, IgG, IgG1, and IgG2) in people exposed to DENV. We observed a significant difference in anti-aGal IgG and IgG1 levels among dengue severity classifications. Furthermore, a significant positive correlation was observed between the anti-aGal IgG and the number of days with dengue symptoms in patients. Additionally, both anti-aGal IgM and IgG levels differ between the two geographical locations of patients. While the anti-aGal IgM and IgG2 levels were not significantly different according to the dengue severity levels, age was negatively correlated with anti-aGal IgM and positively correlated with anti-aGal IgG2. Significant involvement of aGal antibodies in Dengue infection processes is suggested based on the results. Our results open the need for further studies on the exact roles and the mechanisms of the aGal antibodies in Dengue infection.

## Introduction

Dengue virus (DENV) from the Flaviviridae family is transmitted by *Aedes (Ae.)* mosquitoes – mainly *Ae. aegypti and Ae. albopictus* (1). Dengue fever is an infectious disease caused by any of the four DENV serotypes (DENV-1, DENV-2, DENV-3, and DENV-4) (2). Dengue fever has become one of the fastest-growing reemerging mosquito-borne diseases on the planet (3) with a 30-fold surge in the last five decades. The spread of this disease has been linked to population increase, urbanization, and climate change in more than 100 countries in the Asia-Pacific region, the Americas, the Middle East, and Africa (4–7). In South America, Colombia is one of the countries with the highest rates of dengue transmission, with all four serotypes circulating and DENV-1 and DENV-2 being the most common (8–11). As a result, Colombia is regarded as being a DENV hyperendemic area.

The most effective control measures for reducing vector-borne diseases to date have been the use of vector control tools such as pesticides, physical devices (*i.e.*, bed nets) and Wolbachia-based mosquito control strategy to reduce both mosquito life span and pathogen transmission (12, 13). However, the challenge of vector control sustainability, as well as the inadequacy of this intervention to reduce dengue infection burden, has prompted the development of additional disease control strategies such as vaccines (14). Due to the presence of several dengue serotypes, cross-reactivity with other flaviviruses, and antibody-dependent enhancement (ADE), the development of a dengue vaccine has been challenging (15), thereby making it difficult to control the spread of dengue fever with vaccination.

A study of N-glycomics of serotype 2 DENV, produced from mosquito cell, found various glycans including galactose-alpha-1,3-galactose (alpha-Gal, hereafter, aGal) (16) which is also found in many important pathogens including several arthropod-borne pathogens, *leishmania* (17), *Trypanosoma* (18, 19), *Borrelia* (20), and *Plasmodium* spp (21). The aGal is a commonly found glycan in mammals except in old-world primates and human. Loss of alpha 1,3-galactosyltransferase enzyme in humans and old-world primates, lacking the endogenous terminal carbohydrate linkage of the aGal trisaccharide (22), allows production of antibodies against the exogenous aGal presented in the gut microbiota and often in the pathogens described above. The human immune system produces aGal-specific natural antibodies, which account for up to 1-5% of circulating IgM and IgG and 0.1-0.2% of serum immunoglobulin (23, 24). The roles of aGal antibodies in the processes of pathogen infections have been studied for infections of the *leishmania*, *Tripanosoma*, and *Plasmodium* spp (17–21, 25–27), but is lacking in DENV infection.

We evaluated the association of DENV infection with levels of anti-aGal IgM, IgG, and two IgG subclasses; IgG1, which plays a key role in eliciting antibody responses against viral infections, and IgG2, responsible for anticarbohydrate IgG responses. Data from a cohort of 75 febrile patients, recruited in Norte de Santander, Colombia in 2020 was examined in this study, and suggests significant roles of anti-aGal in DENV infection.

## Materials and methods

### Ethical considerations

The protocols and methods for this study were reviewed and approved by the Kansas State University Ethics Review Board (IRB#8952, approval date- 10/11/2017). The Cúcuta and Ocaña Hospital Board also approved the methods and the performance of the study in their institutions. Before sample collection, each potential participant (adults, guardians, or parents of minors) was given a thorough explanation of the study’s objectives, and written informed consent was obtained from individuals willing to participate. Blood samples were collected in compliance with the regulations on ethics of research in human participants for Colombia and the United States.

### Experimental design

The focus of the study was to determine the association between dengue fever and IgM, IgG, IgG1, and IgG2 antibody responses to aGal. The variation in aGal antibody titers in participants from a dengue-endemic area in Norte de Santander were studied in terms of their infective status detected by dengue IgM testing, location, days of symptoms, and dengue disease severity classification.

### Study participants and sample preparation

Samples were collected from a total of 75 febrile patients tested for dengue infection using the DENV [NS1]-based IgM ELISA (DENV-IgM) and 10 people from the same region who were asymptomatic (healthy) with unknown dengue status (5 males and 5 females, age range between 19-42 years old). Dengue participants were categorized into the patients showing typical dengue symptom without warning signs, with warning signs, and severe dengue symptom according to the WHO dengue fever classifications (5). A questionnaire was used to record participants demographics such as age, gender, and place of residence. All patients reported that their cases were the first time being diagnosed with dengue fever, indicating primary infections.

Five milliliter blood samples were collected in dry tubes from all volunteers who reported DENV-like symptoms and within 3 to 15 days of fever seeking medical care at the Hospital Universitario Erasmo Meoz in Cúcuta and the Hospital of Emiro Quintero Cañizares in Ocaña (**Supplementary Figure 1**). Samples were tested for DENV using either DENV NS1-based IgM ELISA, (Xerion—IMEX group, Bogota) following manufacturers’ guidelines. Serum was collected and stored at -20°C until it was shipped to the United States for analysis.

### Determination of antibody titers against aGal

Enzyme linked immunosorbent assay (ELISA) procedures were optimized using checkerboard titration for standardization of each antibody including a positive control. The levels of human anti-aGal antibodies were detected using ELISA high absorption capacity polystyrene microtiter 96 well plates coated with 5µg/ml of Gal-alpha 1-3 Gal β1-4 GlcNAC conjugated to human serum albumin (14 atom spacer, Product Code: NGP3334) (Dextra, Shinfield, UK) in 100 μL/well in carbonate-bicarbonate buffer (5mM Na_2_CO_3_ (Acros Organics AC123670010) and 45mM NaHCO_3_ (Sigma-Aldrich S5761). After overnight incubation at 4°C, coated plates were washed 3 times with 100 μL/well PBS containing 0.05% Tween 20 (PBST) (Sigma-Aldrich P3563), blocked with 100 μL/well of 1% bovine serum albumin (BSA) (ChemCruz Cat# sc-2323) in PBST (Sigma-Aldrich) overnight at RT and then washed 3 times with 100 μL/well of PBST. Human serum samples were diluted 1:50 in PBST with final 1% BSA and 100 μL were added into the wells of the antigen-coated plates and incubated overnight at 37°C. Plates were washed 3 times with PBST and treated with 100 μL/well of detection antibodies; goat anti-human IgM conjugated to horseradish peroxidase (mu chain specific, Abcam cat# ab97205), goat anti-human IgG (IgG (H+L) specific, JacksonImmunoResearch Code# 109-035-088), mouse anti-human IgG1 (Fc-specific, SouthernBiotech Cat# 9054-05), or mouse anti-human IgG2 (Fd-specific, SouthernBiotech Cat# 9080-05). Secondary antibodies diluted 1:10,000 for IgM, 1:1000 for IgG and IgG2, 1:100 for IgG1 v/v in blocking solution were added and incubated overnight at room temperature (RT). Plates were washed 3 times with 100 μL/well of PBST and 100 μL/well of 3,3’, 5,5’-Tetramethyl-Benzidine substrate solution (Thermo Scientific, 34022) was added and incubated for IgM (5 min), IgG (15 min), IgG1 (40 min), IgG2 (25 min) at RT. Finally, the reaction was stopped with 100 μL/well of 1M phosphoric acid (H_3_PO_4_) (prepared using Fisher Scientific Cat# 031101), and the optical density (OD) was measured in a spectrophotometer at 450 nm.

Antibody levels were expressed as the ΔOD value: *ΔOD = ODx-ODb* where ODx represents the mean of individual OD in two technical replications and ODb the mean of the blank wells. One spike control on each plate was used for normalization of plate-to-plate variations. Assay variation among samples (inter and intra assay) tested in the study was below 20% and it was only included in the analysis of serum samples with a coefficient of variation ≤ 20% between duplicates.

### Statistical analysis

The ELISA OD values at 450 nm were compared between dengue IgM results, dengue fever classification, participants location of residence, and age by pairwise comparisons using the nonparametric Mann-Whitney U test (p = 0.05). Multiple data points in aGal antibodies in each Dengue severity level were compared by two-way ANOVA test, the test known to be highly tolerant for data sets with violation of normality (28, 29). Spearman’s rank correlation test was used to assess the strength and significance of a relationship between two independent variables. GraphPad Prism, version 9.2.0, (GraphPad Software Inc., La Jolla, CA) was used for all statistical analyses.

## Results

### Cohort characteristics

A total of 75 DENV-infected patients who visited two different levels of Colombia healthcare facility in 2020 were included in the cohort: 53.3% (40) patients from Cúcuta and 46.7 (35) from Ocaña, two cities in Colombia 201.2 km apart each other (**Supplementary Figure 1**). Testing for DENV IgM is a diagnostic tool for identifying dengue infection in its active stage.: According to the test, 67.5% (21) of Cucuta patients were IgM+, and 68.6% (24) of Ocaña patients were IgM+. When it comes to dengue fever, 42.5% (17) of patients without warnings, and 57.5% (23) of patients with warnings visited the Cúcuta health facility, while 31.4% (11) of patients without warnings, 54.3% (19) of patients with warnings, and 14.3% of patients exhibiting severe dengue, visited the Ocaña health facility. The cohort’s age ranged from 0 to 86 years, with a median age of 14 years. The patients experienced dengue symptoms for periods ranging from 2 to 15 days with median day of 6 days (**Table 1**).

**Table 1:** Cohort characteristics by hospital level.

Location (Hospital level)	Total n	DENV IgM		Dengue fever classification			Age Median (range)	Symptom daysMedian (range)
		IgM+	IgM-	w/o warnings	w/warnings	Severe		
Cúcuta (3) Ocaña (2)	40 35	21 24	19 11	17 11	23 19	0 5	14.5 (0-86 years) 13 (3-62 years)	5 (2-15 days) 7 (3-15 days)
Total	75	45 (60%)	30 (40%)	28 (37%)	42 (56%)	5 (6.7%)	14 (0-86 years)	6 (2-15 days)

### High levels of anti- aGal immunoglobulins in patients with DENV positive IgM

**Figure 1:**
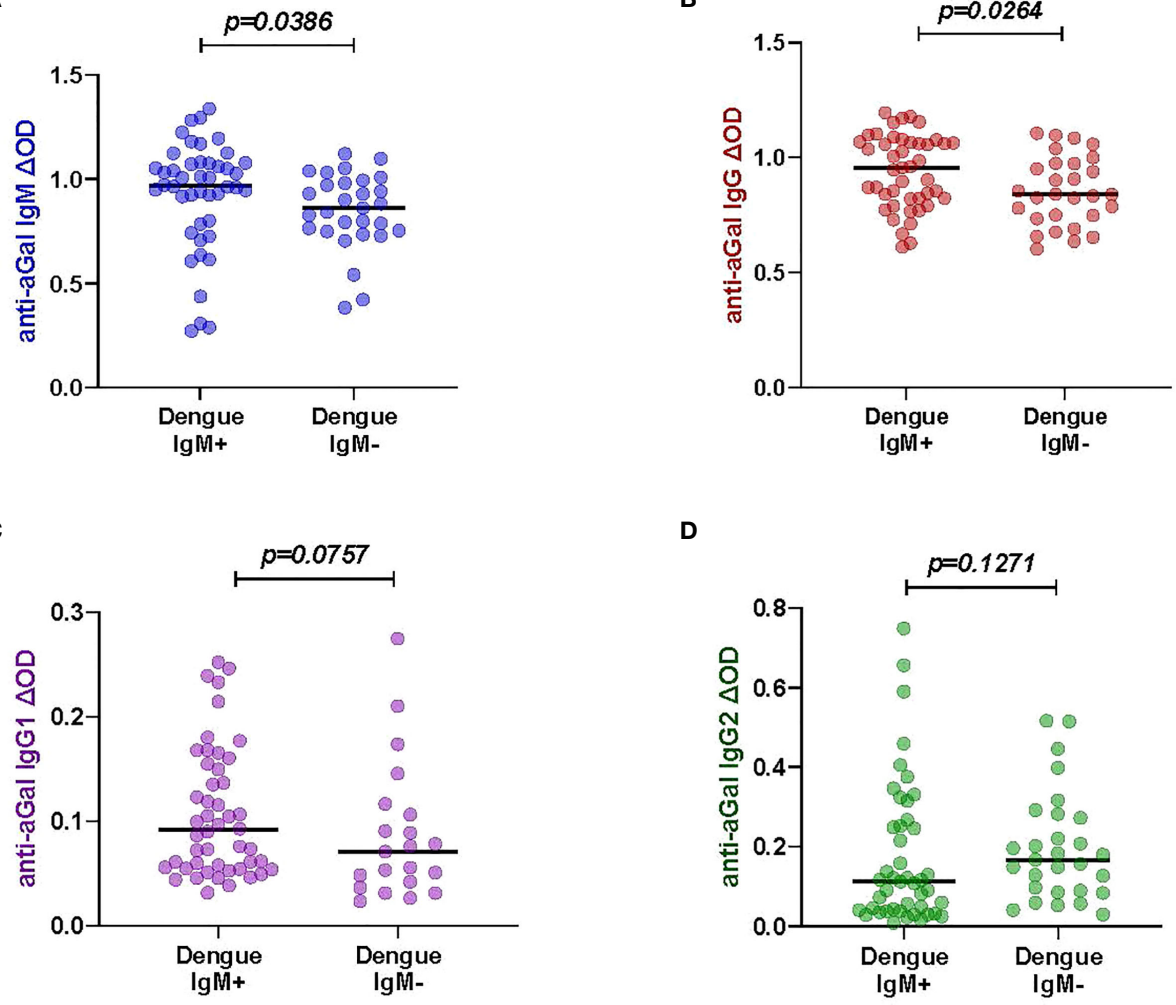
Specific anti- aGal IgM, IgG, IgG1 and IgG2 in dengue patients between Dengue-IgM+ (n=45) and Dengue-IgM- (n=30). **(A)** Blue color - anti-aGal IgM antibody levels. **(B)** Brown color - anti-aGal IgG antibody levels. **(C)** Purple color - anti-aGal IgG1 antibody levels. **(D)** Green color - anti-aGal IgG2 antibody levels. Individual anti-aGal antibody levels are represented by the colored dots and horizontal red lines represent medians of group individual antibody responses Antibody levels are measured in units of OD (optical density). “p” values were based on the pairwise non-parametric Mann-Whitney test.

Measuring the levels of anti-aGal specific IgM, IgG, IgG1, and IgG2 in 45 DENV-IgM (IgM+) and 30 DENV-IgM negative (IgM-) febrile individuals, we observed that DENV-IgM+ patients had significant higher anti-aGal IgM (p = 0.0386) and IgG (p = 0.0264) antibodies than those in DENV-IgM- patients. No significant differences in their anti-aGal IgG1 (p = 0.0757) and anti-aGal IgG2 (p = 0.1271) titers (**Figure 1**) were observed between dengue IgM+ and IgM- patients, although a moderately higher level of IgG2 in IgM- group was observed in IgM- group without statistical significance.

### Positive correlation between anti-aGal IgG and IgG1 levels and Dengue fever severity classification

**Figure 2:**
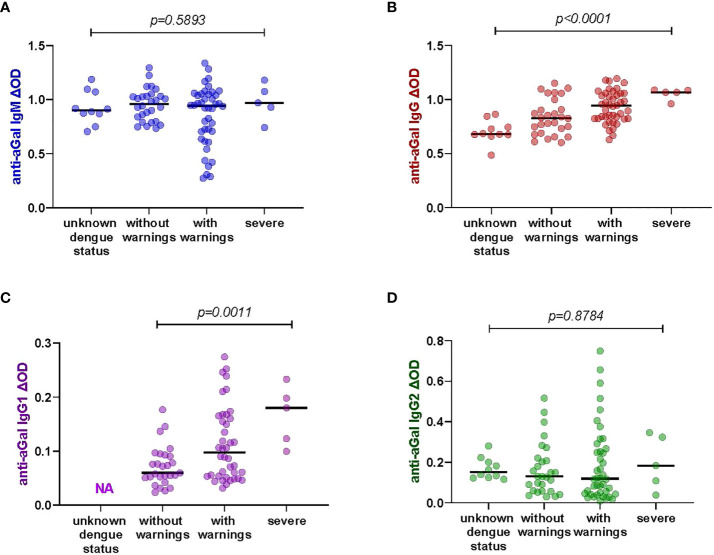
Specific anti-aGal IgM, IgG, IgG1, and IgG2 levels by dengue fever classification; those with unknown dengue status (n-=10), dengue without warnings (n=28), dengue with warnings (n=42), and severe dengue (n=5). **(A)** Blue color - anti-aGal IgM antibody levels. **(B)** Brown color - anti-aGal IgG antibody levels. **(C)** Purple color - anti-aGal IgG1 antibody levels. **(D)** Green color - anti-aGal IgG2 antibody levels. Individual anti-aGal antibody levels are represented by the colored dots and horizontal black lines represent medians of group individual antibody responses. Antibody levels were measured in units of OD (optical density). “p” values <0.05 were measured using the two-way ANOVA test.

We had different categories of dengue severity in our study sample: 28 patients presented dengue without warning symptoms, 42 patients presented dengue with warning symptoms while only 5 had severe dengue symptoms. We included 10 healthy participants with unknown dengue status in this analysis. We tested whether the levels of anti-aGal antibodies was different between the four clinical classifications of dengue (**Figure 2**). We observed that the levels of anti-aGal antibodies, anti-aGal IgG and IgG1, are significantly different depending on the dengue severity (p <0.0001, df =3, F value = 10.65). Further analysis with Mann-Whitney test revealed healthy participants with unknown dengue status had lower anti-aGal IgG than dengue patients without warnings (p = 0.0337), dengue patients with warnings (p = 0.0001), and severe dengue patients (p = 0.0007). The patients without warning symptoms had lower levels of anti-aGal IgG when compared to the with warnings patients (p = 0.0231) or compared with severe patients (p = 0.0079). Furthermore, anti-aGal IgG was also lower in with warnings patients when compared with severe patients (p = 0.0409).

Similar to IgG, anti-aGal IgG1 was also lower in without warnings patients when compared to the with warnings patients (p = 0.0196) and severe patients (p = 0.0004), although this trend between the patients with warnings and severe dengue patients was not significantly different (p = 0.0732). However, we find that anti-aGal IgM and IgG2 levels were not significantly different among the dengue fever classifications and participants with unknown dengue status (anti-aGal IgM p = 0.5740, df = 3, F value = 0.6473; anti-aGal IgG2 p = 0.7249, df = 3, F value = 0.2302) in the two-way ANOVA test. The data for anti-aGal IgG1 in healthy participants with unknown dengue status were not included due to shortage of these serum samples (**Figure 2**).

### Different anti-aGal IgG and IgG1 antibody levels depending on the locations with different hospital levels

**Figure 3:**
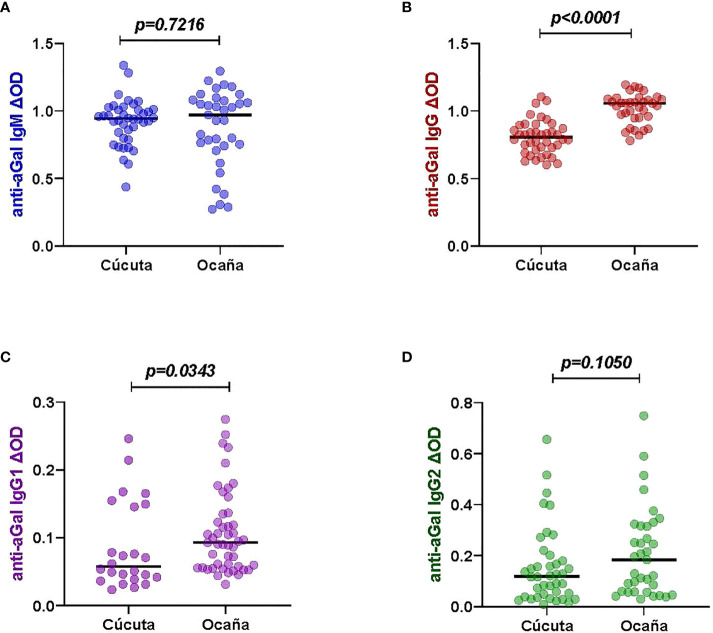
Specific anti-aGal IgM, IgG, IgG1 and IgG2 levels by patient locations with different hospital levels; Cúcuta (n=40) and Ocaña- (n=35). **(A)** Blue color - anti-aGal IgM antibody levels. **(B)** Brown color - anti-aGal IgG antibody levels. **(C)** Purple color - anti-aGal IgG1 antibody levels. **(D)** Green color - anti-aGal IgG2 antibody levels. Individual anti-aGal antibody levels are represented by the colored dots and horizontal black lines represent medians of group individual antibody responses. Antibody levels are measured in units of OD (optical density). “p” values <0.05 were measured using the pairwise non-parametric Mann-Whitney test.

We compared the patients from two different locations- Cúcuta and Ocaña (**Supplementary Figure 1**). The two hospitals in each region were vary in their levels of patient care. Norte de Santander’s capital, Cúcuta, has a level 3 hospital where patients with severe diseases are transferred from level 1 (basic) or level 2 (intermediate) facilities, whereas Ocaña, the department’s second-most populous city with fewer densely populated areas, has a level 2 hospital where patients with mild diseases can seek health care. Significant differences were observed in the anti-aGal antibodies. Among 40 patients from Cúcuta and 35 patients from Ocaña, the patients from Ocaña had higher anti-aGal IgG (p <0.0001) and IgG1 (p = 0.0343) levels than those from Cúcuta (**Figure 3**). In the case of anti-aGal IgM (p = 0.7216) and IgG2 (p = 0.1050), the levels of antibodies were not significantly different between the two locations.

### Positive correlation between anti-aGal IgG and days with dengue symptoms

**Figure 4:**
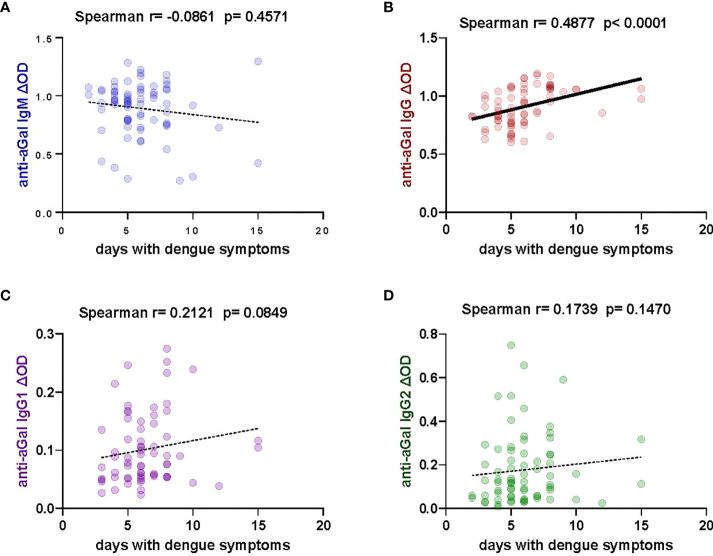
Correlation of specific anti- aGal IgM, IgG, IgG1, and IgG2 antibody levels and days with dengue symptoms in patients. **(A)** Blue color - anti-aGal IgM antibody levels. **(B)** Brown color - anti-aGal IgG antibody levels. **(C)** Purple color - anti-aGal IgG1 antibody levels. **(D)** Green color - anti-aGal IgG2 antibody levels. Regression lines in each graph are shown by solid lines for p<0.05 and dotted lines for non-significant correlations. Antibody levels are measured in units of OD (optical density). “r” and “p” values were measured using the pairwise non-parametric Spearman correlation test.

We investigated the levels of anti-aGal antibodies in relation to the number of days patients presented with dengue symptoms to provide insight into the possible influence of symptom duration on anti-aGal IgM, IgG, IgG1, and IgG2 levels (**Figure 4**). We observed that IgG anti-aGal antibodies showed a significant positive correlation with days with dengue symptoms (Spearman correlation, r = 0.4877; p <0.0001). The levels of anti-aGal-IgM (p = 0.4571) were not significantly affected by the number of days with dengue symptoms. Positive correlations without statistical significance were observed in anti-aGal-IgG1 (p = 0.0849) and anti-aGal-IgG2 (p = 0.1470).

### Dengue fever patients’ age affects anti-aGal IgM and IgG2 levels

**Figure 5:**
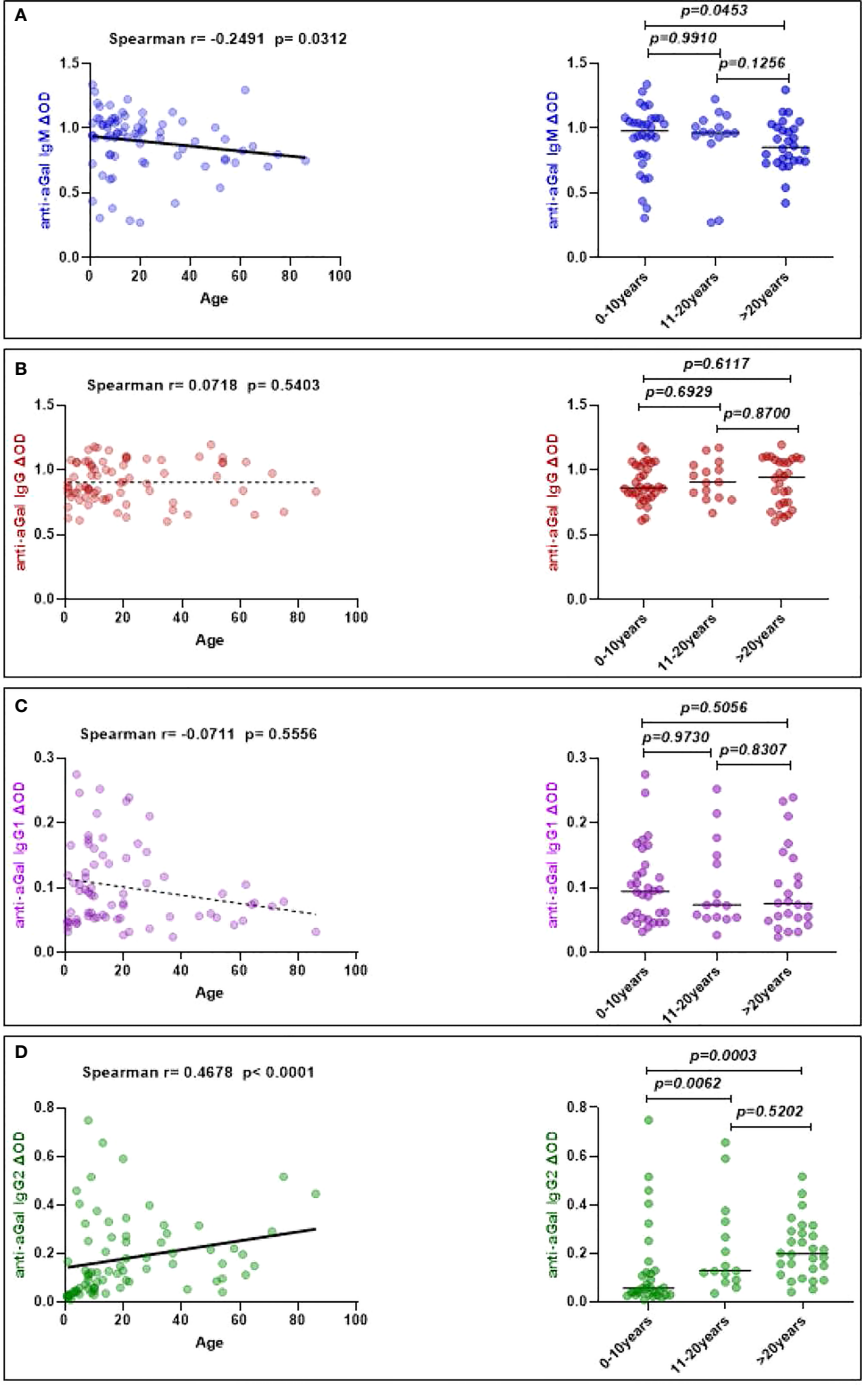
Correlation of specific anti- aGal IgM, IgG, IgG1, and IgG2 antibody levels with dengue patients’ age. **(A)** Blue color - anti-aGal IgM antibody levels. **(B)** Brown color - anti-aGal IgG antibody levels. **(C)** Purple color - anti-aGal IgG1 antibody levels. **(D)** Green color - anti-aGal IgG2 antibody levels. The left panel represents a correlation plot of anti-aGal antibodies with age. Regression lines in each graph are shown by solid lines for p<0.05 and dotted lines for non-significant. Both “r” and “p” values were obtained from the non-parametric Spearman correlation test. While right panel represent anti-aGal antibody with age distribution (0-10 years (n=32), 11-20 years (n=15), and >20 years (n=28)). Anti-aGal antibody levels are represented by the colored dots and horizontal black lines represent medians of group individual antibody responses. The “p” values <0.05 were measured using the non-parametric Mann-Whitney test.

Anti-aGal IgM was negatively associated with age (Spearman correlation, r = -0.2491; p = 0.0312), while anti-aGal IgG2 was positively associated with age (r = 0.4678; p < 0.001), but neither anti-aGal IgG (r = 0.0718; p = 0.5403) nor anti-aGal IgG1 (r = -0.0711; p = 0.5556) were significantly correlated to patients age in the non-parametric Spearman correlation test (**Figure 5**). Further analysis using the Mann-Whitney test (right panel) for the data categorized by age revealed that 0-10 year old patients’ anti-aGal IgM levels were not significantly different when compared to those aged 11-20 years (p = 0.9910) Additionally, anti-aGal IgM levels in the 11-20 year old group were also not significantly different when compared with those patients above 20 years (p = 0.1256), but 0-10 years anti-aGal IgM were significantly higher when compared with those in above 20 years (p value = 0.0453). In the case of anti-aGal IgG2, the 0-10 years dengue patients had significantly lower levels when compared with 11-20 years (p = 0.0062) and above 20 years (p = 0.0003) but 11-20 years anti-aGal IgG2 was not significantly different when compared with above 20 years (p = 0.5202) (**Figure 5**).

## Discussion

Over the decades, dengue has become a major threat to human life in its endemic countries. Colombia has suffered five major dengue outbreaks in the years 1998, 2002, 2010, 2013, and 2019 with the 2010 outbreak being the worst dengue epidemic in the history of the country (11, 30). The DENV envelope protein is an important component needed for the virus to fuse with the cell endosomal membrane and delivery of viral genome into the cytosol (31). Recently, Lei and colleagues identified five types of N-glycan including mannose, GalNAc, GlcNAc, fucose, and sialic acid having high mannose-type N-linked oligosaccharides and the galactosylation of N-glycans on DENV-2 produced in *Ae. albopictus* cell line C6/36 (16). The same study also identified aGal existing in the glycan profile of DENV-2 (16). The carbohydrates of DENV, including aGal, may be important in the process of virus infection and also in the early phase of immune responses of the host although prevalence of aGal in different serotypes of DENV is yet unknown.

The aGal in DENV is likely a specific glycan of the virus produced from the mosquito salivary glands (16), but lacking in the DENV amplified in the human after the first wave of infection. The enzyme for production of aGal, alpha 1,3-galactosyltransferase, has not been identified in arthropod genomes in the homology-based searches (32–34), while multiple copies encoding alpha 1,4-galactosyltransferase and beta 1,4-galactosyltransferase were proposed to be the functional alpha 1,3-galactosyltransferase for production of aGal in arthropods (32, 34). Considering that humans have a pseudogenized alpha 1,3-galactosyltransferase copy and are unable to produce aGal (22), the importance of aGal in DENV infection is likely determined by the aGal antibodies induced at the early DENV infection phase or pre-existing endogenously. Therefore, aGal antibody may be involved in the initial infection of DENV injected with mosquito saliva in dermal and epidermal (keratinocytes and fibroblasts) and skin resident immune cells, and spillover into the bloodstream (Langerhans cells, dendritic cells, and macrophages) (35).

We found that dengue IgM positive (+) patients with active infection presented significantly higher levels of both anti-aGal IgM and anti-aGal IgG (**Figure 2**). The anti-aGal IgG and IgG1 levels were correlated with severe symptoms (**Figure 3**) and anti-aGal IgG with days after the onset of the symptoms (**Figure 5**). Although the data suggests that anti-aGal antibodies play significant roles in DENV infection, the consequences of anti-aGal antibody induction by DENV is yet difficult to predict. Two potential roles of aGal antibody resulting in two opposing effects could be; the role as the protective antibody against the DENV infections and the positive role in antibody-dependent enhancement (ADE) in DENV infection. These potential opposing roles are not necessarily mutually exclusive because they could be antibody-titer dependent in the complex infection processes (36). Concentration-dependent roles of the anti-aGal antibodies in isolated systems, in addition to the current data from DENV patients, would be required to draw a conclusion.

A number of studies have shown that anti-aGal antibodies are neutralization antibodies against pathogens, such as *Plasmodium* spp (27) *Leishmania* spp (17) and *Trypanosoma cruzi* (37) with increased aGal antibody levels upon infection. Moreover, human anti-aGal antibodies were proposed to be the protective antibody against C-type retroviruses carrying aGal, derived from animal cells (38). Although the mechanism is not yet fully understood, aGal immunogen has also shown that it could act as an adjuvant (39–42). In field studies, patients with *Plasmodium vivax* infections had greater anti-aGal IgG and IgM levels than healthy people (43) like the case of DENV infection in this study. Trypanosomiasis and Leishmaniasis also have been associated with elevated anti-gal antibody levels (44). In contrast, patients with *Plasmodium falciparum* infections had lower anti-aGal IgG and IgM levels (21, 45), and patients with *Mycobacterium tuberculosis* infections likewise had lower anti-aGal IgG and IgM levels (45). Although the neutralization activities of the anti-aGal antibody activities were shown in cell line studies for the above pathogens (17, 27, 33), interpretation of population studies for dynamics of anti-aGal antibodies appear to be more complicated.

In our study, higher levels of anti-aGal IgG levels in patient exhibiting severe symptoms (**Figure 3**) and increased levels over the duration of the symptom (**Figure 4**) supports that the anti-aGal IgG could be the antibody induced by the infection with DENV. This delayed anti aGal IgG productions is likely raised by the first wave infection of DENV injected through mosquito saliva, which could carry the immunogenic aGal. Therefore, the increased anti aGal IgG levels exhibited by the DENV infection may function as a protective antibody like the cases in infections of other arthropod borne pathogens, although the host-dependent aGal production in virus is distinguished from autonomous aGal production in other pathogenic organisms. The delayed anti-aGal antibody induction, increased after the expected first wave of viremia, suggests that the increased anti-aGal IgG would function as a protective antibody only against the DENV transmission through additional mosquito bites, which is likely common in a Dengue hyperendemic area.

On the other hand, the anti-aGal IgG may function in the process of ADE in DENV infection. ADE occurs in hosts who already have antibodies reactive to DENV for enhanced infection of the virus into host cells through virus-antibody-Fcγ binding and results in severe dengue symptoms (36). The two opposing roles of the antibodies, neutralization or ADE, are likely determined by the antibody titer (36), specificity of the antibody toward certain protein/residues of the virus (46), and possibly divided roles in different types and subtypes of immunoglobulin. A study for evaluating titer-dependent roles of aGal antibodies in dengue infection would require the data for the anti-aGal immunoglobulin levels in the pre-DENV exposure of the same subjected patient. Furthermore, the roles in ADE for different types and subtypes of immunoglobulin need to be investigated because the significant associations between severe symptoms and high levels of IgG and IgG1 subtypes, combined with healthy individuals exhibiting the low levels (**Figure 2**), support the roles of anti-aGal IgG and IgG1 for ADE. Our study involves only patients who are categorized as first infection while ADE is described for the severe dengue cases in second infections. Therefore, the enhancement role of aGal antibodies in initial DENV entry to host cells, if it is the case, could be determined by pre-exposure to immunogenic aGal through various routes; tick bites, potentially mosquito bites with or without pathogens such as malaria plasmodium, and the microflora and diet in the digestive system and milk.

Geographical differences in anti-aGal antibodies were observed. Both Cúcuta and Ocaña were among the most endemic cities for DENV in the country (47) (**Supplementary Figure 1**). Cúcuta, the capital of Norte de Santander is the most urban settlement with a level 3 health facility where severe patients are referred to from level 1 (basic) or level 2 (intermediate) health care facilities whilst Ocaña is an area with less urbanization than Cúcuta with a level 2 hospital where patients with mild disease seek health care (48, 49). It was interesting that both healthcare facility patients had distinct levels of anti-aGal IgG and IgG1, albeit the 5 severe cases were all from the Ocaña healthcare facility. This further strengthens the assumption that anti-aGal IgG and IgG1 might be associated with the progression of dengue disease.

Our study indicated that anti-aGal IgM was negatively, and anti-aGal IgG2 was positively correlated with individuals’ ages while age had no significant effects on the anti-aGal IgG and IgG1 levels (**Figure 5**). A previous study described positive associations of both anti aGal IgM and IgG levels with age in infants and children, but without age groups comparable to our study (50). Serum immunoglobulin levels up to 18 years old also showed generally increasing patterns of IgM and IgG over time (50, 51). Interestingly, anti aGal IgE level, the immunoglobulin that is involved in the alpha-gal syndrome (red-meat allergy) (52, 53), was found to be negatively correlated to the anti aGal IgG2 level and positively to that of anti aGal IgG1 (54). Although we do not have the data for IgE levels in this study, comparisons of the levels of different immunoglobulin indicate positive correlations between anti aGal IgM and anti-aGal IgG1, and between anti-aGal IgG1 and anti-aGal IgG (**Supplementary Figure 2**), implying that the levels of anti-aGal IgG2, responsible for anticarbohydrate, may be independently regulated from the levels of anti-aGal IgG1, IgM, and possibly IgE.

An innovation in the evolution of the human immune system includes generation of expanded repertoires of anti-glycan antibodies*, i.e.*, anti-aGal that is associated with the loss of endogenous aGal. Escalation of host and pathogen arms race to involvement of vector in production of aGal on the pathogenic DENV is the case that we examined in this study. Although we were unable to draw a solid conclusion for the roles of the aGal antibodies, whether anti-aGal antibodies are a positive or negative factor in DENV infection, a significant role in the infection process is proposed with the population data showing different levels of anti-aGal antibodies over dengue progressions.

## Data availability statement

The original contributions presented in the study are included in the article/**Supplementary Material**. Further inquiries can be directed to the corresponding authors.

## Ethics statement

The studies involving human participants were reviewed and approved by Kansas State University Ethics Review Board (IRB#8952, approval date- 10/11/2017). Written informed consent to participate in this study was provided by the participants’ legal guardian/next of kin.

## Author contributions

Conceptualization, all authors; sample collection— JC, LG-S, MG, and BL-R; methodology—OO, LM-R, and YP; data analysis—OO and YP; writing—original draft preparation, OO and YP; writing—review and editing, OO, SF, LM-R, YP, and BL-R; visualization, OO, SF, LM-R, BL-R, and YP; supervision, YP and BL-R; project administration, BL-R and YP; funding acquisition, OO, YP, and BL-R. All authors have read and agreed to the published version of the manuscript.

## Funding

This research was funded by the 2021-22 Donald C. Warren Scholarship to OO in K-State, NIH-NIAID R21 AI163423 to YP, and COBRE-NIH 5P20GM103638-08 to BL-R. This study is contribution no. 23-055-J from the Kansas Agricultural Experiment Station.

## Acknowledgments

The authors thank the Kansas State Department of Entomology and the College of Agriculture. We also thank the National Institute of Health for the funding.

## Conflict of interest

The authors declare that the research was conducted in the absence of any commercial or financial relationships that could be construed as a potential conflict of interest.

## Publisher’s note

All claims expressed in this article are solely those of the authors and do not necessarily represent those of their affiliated organizations, or those of the publisher, the editors and the reviewers. Any product that may be evaluated in this article, or claim that may be made by its manufacturer, is not guaranteed or endorsed by the publisher.
