# Supplementary material for: Association of dengue infection with anti-alpha-gal antibodies, IgM, IgG, IgG1, and IgG2
Source: Front Immunol. 2022 Oct 14;13:1021016. doi: 10.3389/fimmu.2022.1021016 (PMC9614307; doi:10.3389/fimmu.2022.1021016)
Supplement: Supplementary file 1 [file DataSheet_1.pdf]

Supplementary Material

1 Study location map

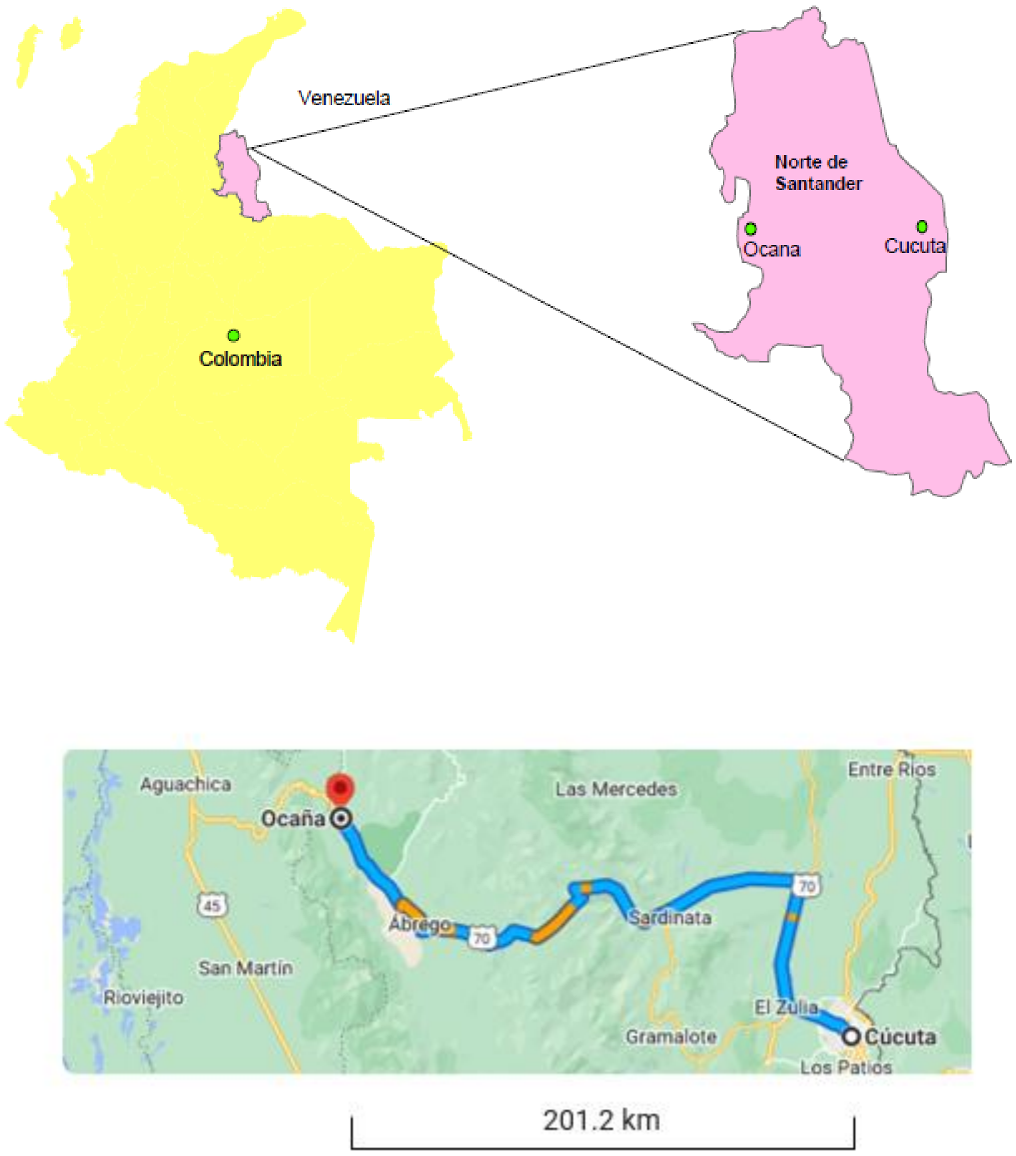

Supplementary Figure 1. Map showing the geographical location of study samples. The yellow map shows Colombia while the pink map shows the two municipalities of Norte de Santander where sera were collected. The green map shows the distance of 201.2 km between the Cucuta and Ocana municipalities. Map created using MindtheGraph, Mapline, and Google.

2 Anti-aGal IgG1 correlates positively with increasing anti-aGal IgM and IgG Supplementary Data

We used the non-parametric Spearman correlation test to assess the different levels of anti-aGal immunoglobulins, and we observed anti-aGal IgM had no significant correlation with anti-aGal IgG (r= 0.1765; p= 0.1221), anti-aGal IgG had no significant correlation with anti-aGal IgG2 (r= 0.1548; p= 0.1760), anti-aGal IgM had no significant correlation with anti-aGal IgG2 (r= -0.1896; p= 0.0964), anti-aGal IgG2 had no significant correlation with anti-aGal IgG1 (r= 0.0926; p= 0.5180) but anti-aGal was having significant positive correlations with both anti-aGal IgM (r= 0.3081; p= 0.0278) and anti-aGal IgG(r= 0.4122; p= 0.0026) (Supp Fig. 1).

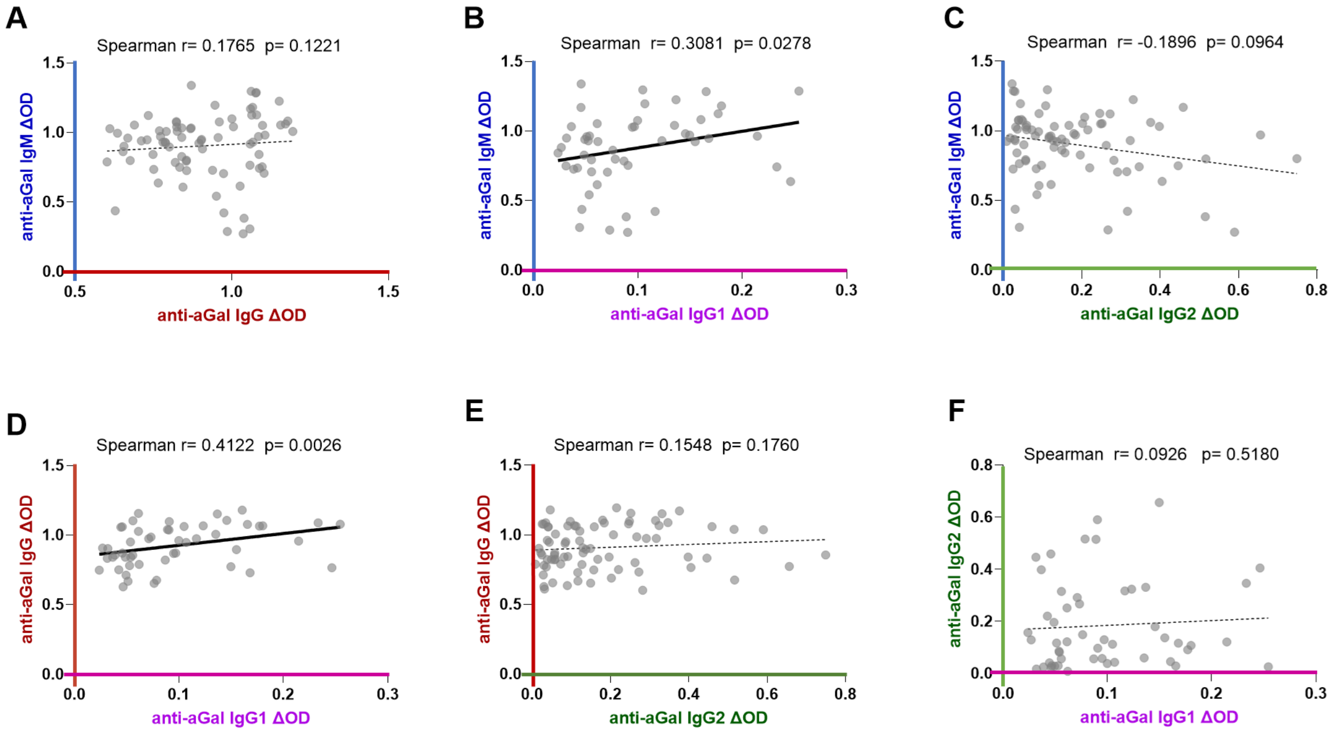

Supplementary Figure 2. Correlation of specific anti-aGal immunoglobulins IgM, IgG, IgG1, and IgG2 antibodies (A) anti-aGal IgM and IgG correlation (B) anti-aGal IgM and IgG1 correlation (C) anti-aGal IgM and IgG2 correlation (D) anti-aGal IgG and IgG1 correlation (E) anti-aGal IgG and IgG2 correlation (F) anti-aGal IgG2 and IgG1 correlation. Regression lines in each graph are shown by solid lines for $p<0.05$ and dotted lines for non-significant correlation. Antibody levels are measured in units of OD (optical density) "r" and "p" values were measured using the pairwise non-parametric Spearman correlation test.
